# A High-Multi Target Resolution Focal Plane Array-Based Laser Detection and Ranging Sensor

**DOI:** 10.3390/s19051210

**Published:** 2019-03-09

**Authors:** Hyeon-June Kim, Eun-Gyu Lee, Choul-Young Kim

**Affiliations:** 1 CIS Development Division, SK Hynix, Icheon 17336, Korea; hyeonjunekkim@gmail.com; 2 Department of Electronics Engineering, Chungnam National University, Daejeon 34134, Korea; eungyu@cnu.ac.kr

**Keywords:** FPA-based LADAR, ToF, multi-target resolution, multiple echo detection

## Abstract

This paper introduces a digital-assisted multiple echo detection scheme, which utilizes the waste time of the full serial data readout period in a focal plane array (FPA)-based laser detection and ranging (LADAR) receiver. With the support of an external digital signal processor (DSP) and additional analog memory inserted into the receiver, the proposed readout scheme can effectively enhance multi-target resolution (MTR) three times higher than the conventional FPA-based LADAR, while maintaining low power consumption and a small area. A prototype chip was fabricated in a 0.18-μm CMOS process with an 8 × 8 FPA configuration, where each single receiver pixel occupied an area of 100 μm × 100 μm. The single receiver achieved an MTR of 20 ns with 7.47 mW power dissipation, an input referred noise current of 4.48 pA/√Hz with a bandwidth 530 MHz, a minimum detectable signal (MDS) of 340 nA, a maximum walk error of 2.2 ns, and a maximum non-linearity of 0.05% among the captured multiple echo images.

## 1. Introduction

Recently, laser detection and ranging (LADAR) systems have been developed due to growing interest from many diverse fields, including automation engineering, military technology, three-dimensional (3D) imaging, geographical mapping, and, especially, range-finding [1,2,3]. LADAR systems can be classified by their detection mechanisms. These classifications include continuous-wave optical phase-based, structured light-based, and short light pulse-based detection sensors [4,5,6]. In particular, the short light pulsed time-of-flight (ToF) LADAR system, which can measure distances to targets using the ToF of emitted and detected laser pulses, is widely used due to its ability to deliver more accurate and denser range measurements, which are independent of natural lighting conditions [7]. Among various readout architectures for short light pulsed ToF LADAR, the focal-plane array (FPA)-based LADAR architecture is suitable for the detection of objects moving quickly in real time [8,9].

**Figure 1:**
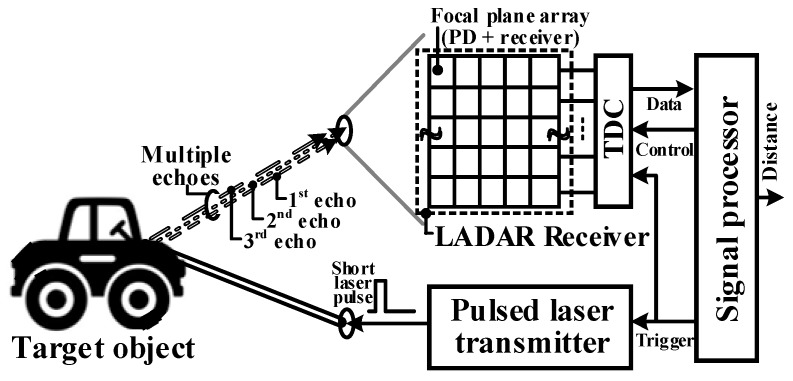
Block diagram of the typical pulsed time of flight (ToF) laser detection and ranging (LADAR) system. PD: photodiode; TDC: time-to-digital converter.

A block diagram of a typical pulsed FPA-based LADAR system is shown in Figure 1. A pulsed FPA-based LADAR system consists of a pulsed laser transmitter, optical LADAR receiver, and signal processing module. A short light pulse is emitted through the transmission optics of the laser transmitter, and the light pulses reflected by the target object are captured by the photodiodes (PDs) of the two-dimensional (2D) FPA in the LADAR system. Note that the PD array is laid on the readout receiver array as a flip-chip bonding structure. The time difference between a transmitted and reflected light pulse detected by the receiver is converted by the time-to-digital converter (TDC) to a digital value, and the associated distance is calculated in the signal processor. As shown in Figure 1, a fraction of the transmitted light pulse is reflected back from the target to the receiver (i.e., single echo), and the returned light pulses have a temporal difference between each other (i.e., multiple echoes) according to the shape or multiple surfaces of the target object [10].

Multi-target resolution (MTR) represents the capability of a LADAR receiver to collect a series of returned light signals (multiple echoes) from the targeted object without the loss of signal information [11]. For reliable data coverage for a distance from the target object, MTR has become an important design consideration. While most of the previous research has been focused on the bandwidth of the transimpedance amplifier (TIA) in the LADAR receiver in an attempt to improve the MTR [8,12], the detection latency induced by the peripheral circuits or readout architecture has been the major cause of a speed bottleneck. An attempt to solve this problem has been to increase the number of receivers in the LADAR system as in [13,14], but occupied area and power consumption of the dedicated circuits are major drawbacks to this approach. In FPA-based LADAR, power management should be considered at a unit pixel level to keep the power consumption to an acceptable level for commercial applications. Moreover, the size of the pixel circuits should be small enough to fit within the required pixel dimension for the resolution of the FPA. This is dictated by the increasing demand for pixel resolution and accurate 3D imaging in commercial LADAR.

With these motivations, this work proposes a pulsed FPA-based LADAR receiver architecture with a digital-assisted multiple echo detection scheme for middle-range detection applications. With the support of an external digital signal processor (DSP) and additional analog memory inserted into the receiver, the proposed single-channel receiver architecture could run as effectively as a three-channel receiver. This results in an enhanced MTR of the receiver approximately three times that of a typical FPA-based LADAR system. In addition, the proposed receiver extracts range information from the target object along with intensity, which carries information about the object’s reflecting surfaces.

## 2. Proposed Readout Scheme

**Figure 2:**
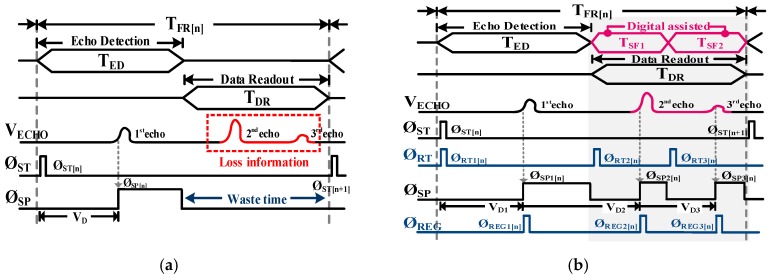
(**a**) conventional readout scheme and (**b**) proposed readout scheme in a focal plane array (FPA)-based LADAR.

A conventional one-channel receiver operates as follows: after the first echo detection, the full readout period involving all the relevant circuits has to be completed in time for the next echo detection. It is thus difficult to detect successively incoming echoes without time delays. In the case of the FPA-based LADAR system, the MTR of the receiver can be determined by its frame rate, consisting of the echo detection time (T_ED_) and the data readout time (T_DR_). The simplified operation timing diagram for the LADAR receiver using the FPA configuration is shown in Figure 2. For a simple explanation, let us assume that the first echo signal is incoming during T_ED_, and the second echo and third echo are incoming during T_DR_. As shown in Figure 2a, after a short light pulse is emitted by the laser transmitter at the Ø_ST[n]_, the receiver detects the first echo at Ø_SP[n]_ during T_ED_, where, n denotes the frame number. Given that all the data from the FPA should be transferred into the analog-to-digital convertor (ADC) during T_DR_, the receiver would be wasting time (T_DR_) waiting for the following synchronized Ø_ST[n+1]_ activation in order to execute the next echo detection. It could result in the loss of echo information (second echo and third echo) within T_DR_ even though the first echo had already been detected at the Ø_SP[n]_. In the case of the serial data readout structure as in [15,16,17], the wasted time (T_DR_) increases if the pixel resolution is increased and makes it difficult to successively detect incoming echoes, resulting in the deterioration of the detection latency of the FPA-based LADAR system. Here, the detection latency means the total time to become ready to receive the next incoming echo after the previous echo detection.

To solve this problem, the proposed readout scheme utilizes the time period of T_DR_ for multiple echo detection with the support of the DSP as shown in Figure 2b. Once the frame rate of the FPA-based LADAR system is set, one frame readout time of the receiver (T_FR[N]_) can be divided into three sub-frames (T_ED_, T_SF1_, and T_SF2_) through the timing controller in the DSP. Note that the timing controller generates T_SF1_ and T_SF2_ evenly within T_DR_. Within the periods of T_SF1_ and T_SF2_, because the receiver could continue to read incoming echoes twice, after Ø_RT2[N]_ and Ø_RT3[N]_, and to store those results in the analog memory built into the receiver at Ø_REG2[n]_ and Ø_REG2[n]_, the throughput of a single-channel receiver could effectively become similar to a three-channel receiver for multiple echo detection. This leads to a reduction in the structural limitation of the FPA-based readout architecture and a tripling of its MTR over the conventional one.

## 3. Proposed LADAR Receiver and Implementation

**Figure 3:**
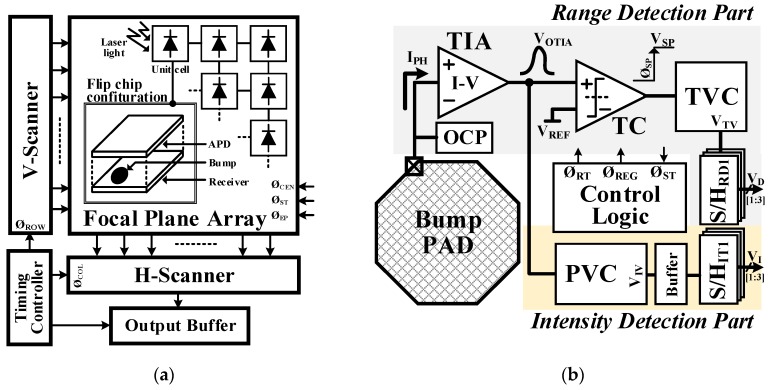
(**a**) overall block diagram and (**b**) simplified block diagram of the proposed LADAR receiver. TIA: transimpedance amplifier; TC: timing comparator; TVC: time-to-voltage converter; PVC: peak-amplitude-to-voltage converter.

The prototype pulsed ToF LADAR architecture is illustrated in Figure 3. It includes an avalanche photo diode (APD) array with a flip-chip bonded receiver array via a metal bump electrical contact, a V-scanner for sequential row activation, an H-scanner for sequential column activation, an output buffer of 50 Ω for output impedance matching, and a timing controller for control signal generation.

The simplified block diagram of the proposed LADAR receiver is shown in Figure 3. The proposed receiver consists of two parts: the range detection-related circuit (RDC) and the intensity detection-related circuit (IDC). The RDC part consists of a transimpedance amplifier (TIA), a timing comparator (TC), a time-to-voltage converter (TVC), and three sample and hold circuits (S/H_RD_) as analog memory and an over current protector (OCP). It detects the range information of the incoming three echoes during the period of one timeframe. The TIA converts the photo current (I_PH_) pulse of the APD into a voltage (V_OTIA_) after the Ø_RT_. The TC compares the V_OTIA_ with the pre-determined threshold voltage (V_REF_) as analog memory. When the rising edge of V_OTIA_ exceeds a certain threshold V_REF_, the TC produces a V_ST_ signal, which indicates the arrival of the returned light signal. The TVC waits for the V_ST_ signal from the TC and a proportionally converts the time difference between the Ø_ST_ and the Ø_SP_ into a voltage, which represents range information (V_TV_). Given that the Ø_REG_ is generated from control logic as a sampling clock signal of the S/H_RD_ on every rising edge of the Ø_SP_ within the one timeframe, the V_TV_s of the incoming three echoes—V_TV[1]_, V_TV[2]_, and V_TV[3]_—can be stored sequentially in the three S/H_RD_s. The OCP circuit protects the APD pixel and the receiver circuit from damage due to exposure to excessive optical power. The IDC part consists of a peak-amplitude-to-voltage converter (PVC), an output buffer, and three sample and hold circuits (S/H_S_). It detects the strength of the incoming three echoes as intensity information. The PVC samples the peak amplitude of the V_OTIA_ before triggering the Ø_REG_ within every sub-frame, and the output buffer drives its results V_IV_ into S/H_IT_.

**Figure 4:**
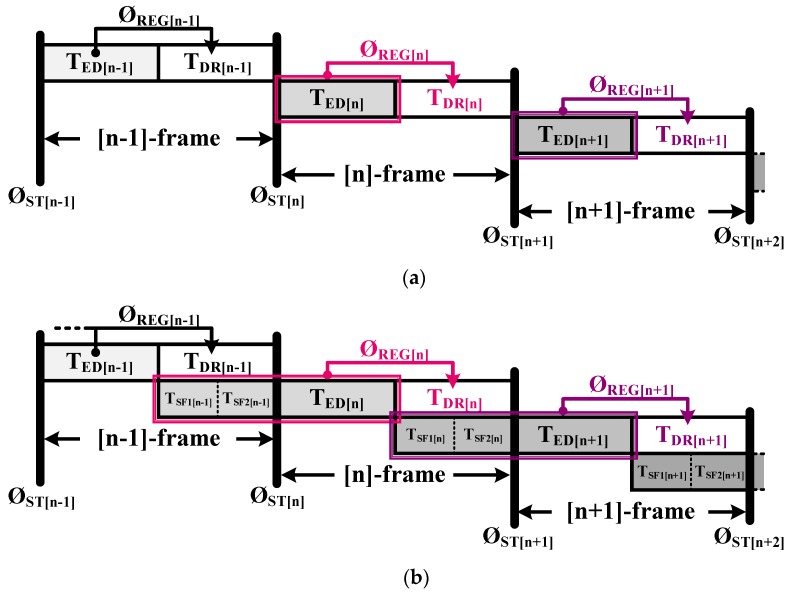
Data readout timing of (**a**) a conventional and (**b**) the proposed LADAR receiver.

Compared with the conventional LADAR receiver, as shown in Figure 4a, in the proposed receiver, the S/H can be used as a timing buffer to allot T_SF1[n−1]_ and T_SF2[n−1]_ in parallel with T_DR_ in order to effectively utilize the operation period in the FPA-based LADAR system. As shown in Figure 4b, because S/H_SF1_ and S/H_SF2_ memorize the previous second echo and third echo information due to time sharing with T_SF1[n−1]_, T_SF2[n−1]_, and T_DR_, all the echo information from T_SF1[n−1]_, T_SF2[n−1]_, and T_ED[n]_ would be read at Ø_REG[n]_ during T_DR[n]_ in the [n]-frame in parallel. After all the data is collected from the receiver array, three range images and three intensity images for T_SF[n−1]_, T_SF[n−1]_, and T_ED[n]_ can be generated and simply rearranged according to the frame number in the DSP without any calibration.

**Figure 5:**
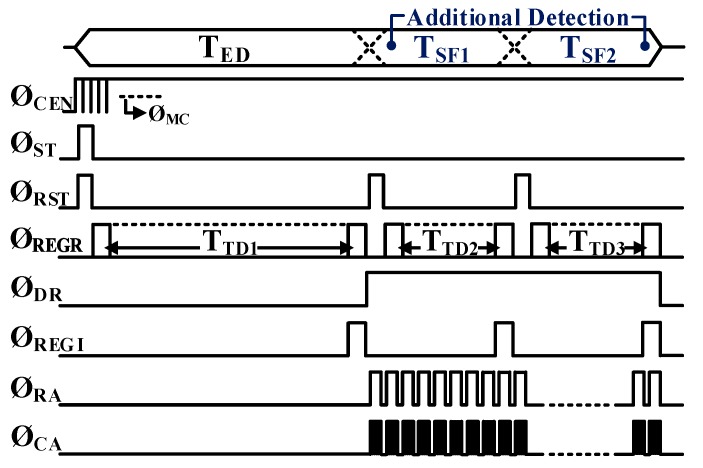
Operational timing diagram of the prototype FPA-based LADAR.

A simplified operational timing diagram of the FPA-based LADAR system, employing the proposed digital-assisted readout scheme, is illustrated in Figure 5. When the chip is enabled by Ø_CEN_, the master clock of 40 MHz (Ø_MC_) starts to trigger the synchronization of all the operational circuits on the chip. The proposed readout scheme utilizes all the operational periods (T_FR_) by inserting additional detection periods (T_SF_) into the waste time, resulting in three sub-frames (T_ED_, T_SF1_, and T_SF2_) for multiple echo detection. Each sub-frame is activated with reset signal triggering (Ø_RST_) during T_FR_. Within each sub-frame, if the returned echo is found during the timing detection period (T_TD_), its range information is stored in the S/H_RD_ with the triggering of the range register (Ø_REGR_). At the same time, the echo intensity detection is performed, and its intensity information is stored in the S/H_IT_ at the rising edge of the intensity register (Ø_REGI_). During the data readout period (Ø_DR_), each row of the FPA is activated in sequence with row readout triggering (Ø_RA_), and all the columns in the selected row are read by the sequential column activation of the V-scanner with column readout triggering (Ø_CA_), as in the serial data readout.

**Figure 6:**
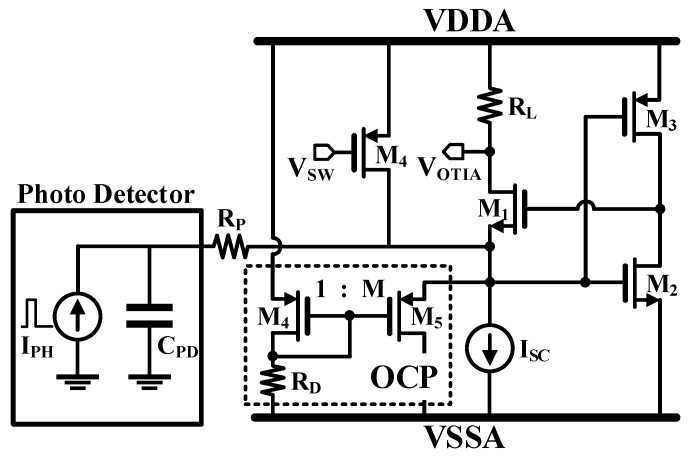
Simplified schematic of a transimpedance amplifier with an over current protector (OCP).

In this work, the TIA has a regulated cascode (RGC) topology based on inverter local feedback for a low input impedance, resulting in wider bandwidth [8], as shown in Figure 6. Here, VDDA indicates the supply voltage and VSSA indicates the ground voltage for the TIA circuit operation. In order to obtain a high dynamic range, the TIA output was designed to drive the signal toward the positive supply rail with the load resistance R_L_. Considering the many functional blocks and shielding lines in the receiver design, one of the challenges is to integrate all of the blocks within the targeted pixel area. The RGC TIA topology is a simple circuit configuration and is suitable to fit into a small APD dimension, even though it inherently has a somewhat high input-referred current noise.

The transimpedance gain (*Z_T_*) and small-signal input impedance (*Z_IN_*) of the TIA can be approximated by:

(1) $$Z_{T}≅R_{L}$$

(2) $$Z_{IN}≅\frac{1}{g_{m1}\left(1+\left(g_{m2}+g_{m3}\right)\left(r_{ds2}//r_{ds3}\right)\right)}$$

where $g_{m}$ and $r_{ds}$ are the transconductance and the output resistance, respectively. The required bandwidth of the TIA can be approximated from [4] as:

(3) $$BW≅\frac{0.35}{t_{r}}$$

where $t_{r}$ is the rise time of the input optical pulse. The full width at half maximum (FWHM) of the input pulse is approximately 3.8 ns and its rise time is approximately 1 ns. The C_PD_ and R_P_ are approximately 1 pF and 100 Ω, respectively.

**Figure 7:**
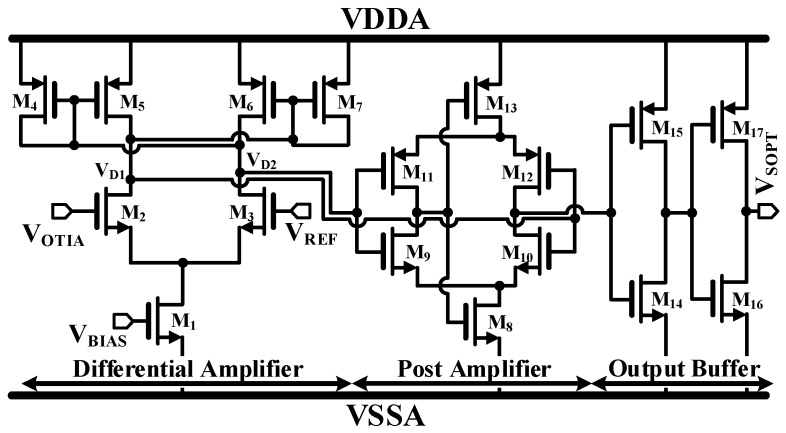
Simplified schematic of a timing comparator.

The TC is designed in three stages: the differential amplifier, the post-amplifier, and the output buffer as shown in Figure 7. The differential amplifier compares the V_OTIA_ with the V_REF_ and transfers the differential signal (V_D1_ and V_D2_) to the post-amplifier, which is a self-biased topology [18]. Positive feedback from the cross-gate connection of V_D1_ through V_D2_ is constituted in order to increase the gain of TC. Two inverters are added as output buffers to isolate the load capacitance with an additional gain.

**Figure 8:**
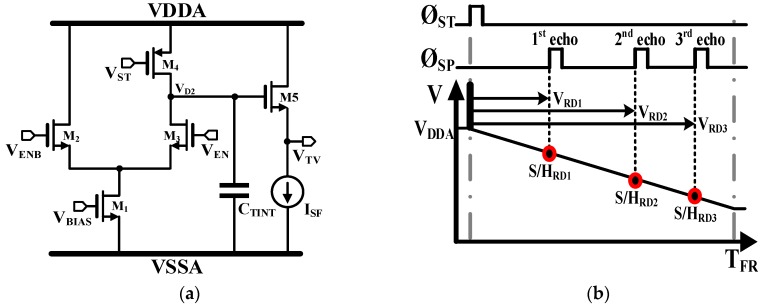
(**a**) Simplified schematic of a TVC and (**b**) its operation.

The schematic of the time-to-voltage converter (TVC) [19] and its operation timing diagram are shown in Figure 8. The TVC converts the time difference between Ø_ST_ and Ø_SP_ into a voltage proportionally as shown in Figure 8b. The complementary inputs V_ENB_ and V_EN_ signals are used to steer the current of M_1_ (I_TV_) closed and opened to pre-charge the integration capacitor (C_TINT_) to V_DDA_.

During V_EN_ enabled, the TVC discharges C_TINT_, while S/H_RD_ samples from M_2_ to M_3_ and then back to M_2_. Initially, the switch V_TV_ is voltage of

(4) $$\Delta V_{TV}≅\frac{I_{TV}}{C_{TINT}}\times \Delta t$$

where Δt is the time interval being measured. In this work, the nominal values for I_TV_ and C_INT_ are 1.6 μA and 456 fF, respectively. I_TV_ has a range varying from a minimum of 0.4 μA to a maximum of 6.4 μA.

**Figure 9:**
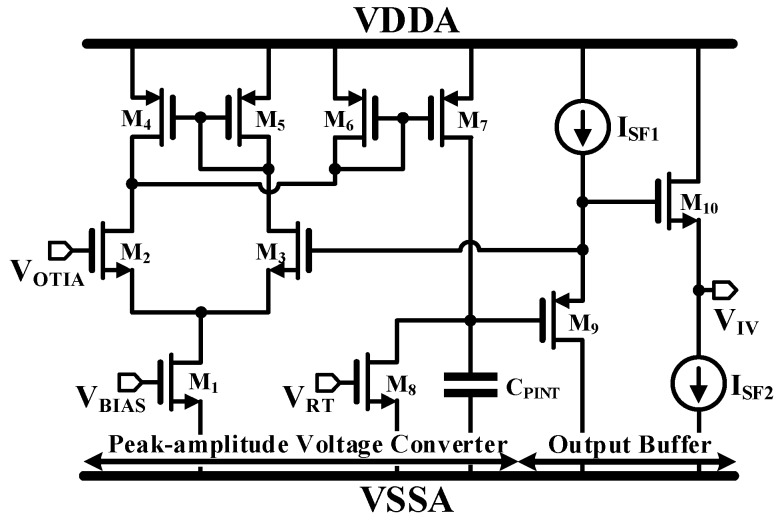
Simplified schematic of a PVC.

The peak amplitude of V_OTIA_ is related to the reflectivity of the target object (i.e., intensity). The PVC thus detects the peak of the returned light pulse as intensity. A conventional structure is used for the PVC [20] as shown in Figure 9. M_1_ through M_8_ constitute the operational transconductance amplifier (OTA) with the rectifying mirror as the peak detector, and M_9_ through M_10_ constitute the inner source follower as the output buffer. After the PVC is reset to the integration capacitor (C_PINT_) with Ø_ST_, when the V_OTIA_ is higher than the voltage of the C_PINT_ (V_PEAK_), which that means V_OTIA_ reaches the highest peak, M_7_ will charge the C_PINT_ until the V_PEAK_ is equal to V_OINT_. On the other hand, when V_OTIA_ becomes lower than V_PEAK_, the OTA switches off the mirror of M_6_ and M_7_.

An excessive photo current can increase the input voltage of the TIA beyond the breakdown voltage [8]. A simple and effective OCP is adopted in the input node of TIA as shown in Figure 6. When the source voltage of M_5_ rises above 1.7 V, the OCP turns on and the size ratio of M_4_ and M_5_ are chosen to safely sink up to several mA of excess current.

## 4. Measurement Results and Discussions

**Figure 10:**
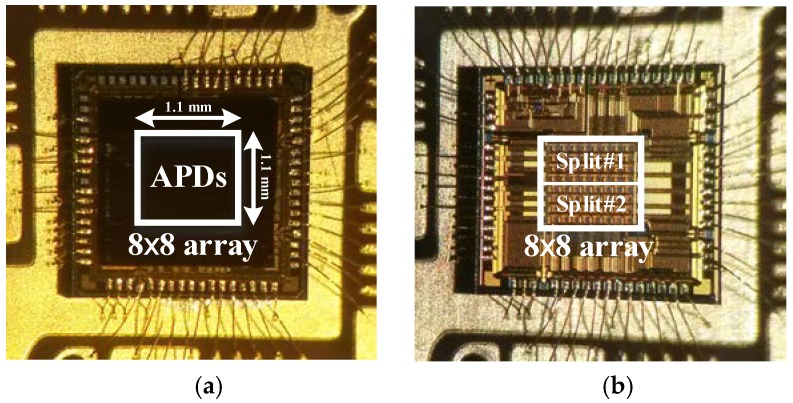
(**a**) Chip microphotograph with flip-chip and (**b**) without flip-chip-bonded avalanche photodiode (APD).

**Figure 11:**
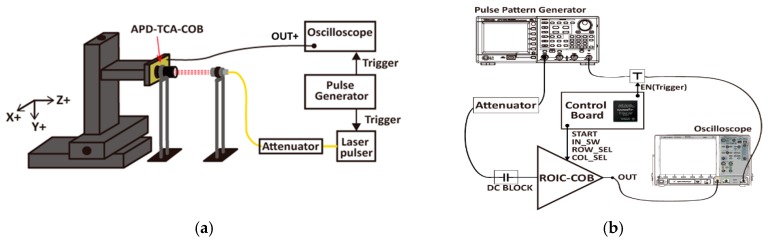
Measurement setup for (**a**) the optical response test and (**b**) the electrical response test.

The prototype chip was fabricated in a 0.18-μm CMOS process. The chip microphotographs of the proposed LADAR receiver are shown in Figure 10. The 8 × 8 array of the prototype photo detector was laid down on the 8 × 8 array of the receiver as a flip-chip bonding structure. The prototype receiver applying the proposed digital-assisted readout scheme was implemented in a chip size of 2.2 mm × 2.2 mm with peripheral circuitry, including I/O pads. The 8 × 8 array of the proposed receiver occupied an area of 1.1 mm^2^ with a unit pixel size of 100 μm × 100 μm. In order to compare the performance of the proposed readout scheme with a conventional one, the prototype chip was implemented in two split patterns: rows 1–4 with conventional readout scheme (Split#1) and rows 5–8 with a proposed readout scheme (Split#2). Note that Split#1 has the same receiver structure as Split#2, but it does not have the analog memory for sharing operation time. Two types of measurements were applied in order to verify the prototype chip: The optical response test (Figure 10a) involved carrying out a measurement using an optical laser pulse with a wavelength of 1550 nm [21], a FWHM of 3.8 ns, and a repetition rate of 62.5 kHz through an attenuator and collimator (as shown in Figure 11a). The electrical response test (Figure 10b) to verify the operation of the receiver only involved concurrently inducing an electrical current pulse with a width of 3.8 ns and a rise and fall time of under 100 ps in all the pixels of the FPA in place of the optical current pulse from the APD (as shown in Figure 11b).

**Figure 12:**
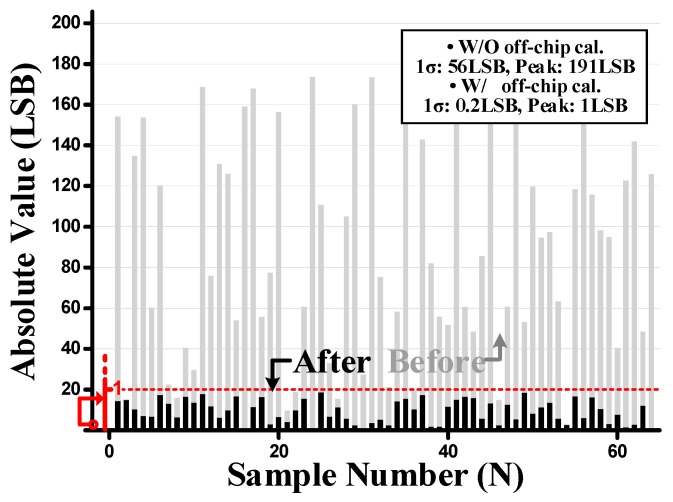
Pixel fixed pattern noise of the prototype chip.

In this work, the InGaAs APD was implemented as a test pattern. The variation between the adjacent APD pixels was thus larger than in the conventional one, resulting in larger fixed pattern noise (FPN) [22], which appeared as a stain in the captured echo image. The APD variation profile was measured from 64 samples with no echo signal, as shown in Figure 12. The horizontal-axis represents the sample number with the vertical axis representing the absolute value of the sample at 10-bit resolution. The measured standard deviation of pixel FPN was 56 LSB, which is approximately 5.4% of the full scale, which has a peak value of 191 LSB. This made it difficult to clarify the distinct range information. In order to reduce the pixel FPN of the prototype APD, off-chip digital offset adjustment was performed as off-chip calibration as in [17,23], so that the offset difference between neighboring pixels could be reduced to under 1 LSB. After offset adjustment, the pixel FPN was reduced to less than 0.1%.

The measured rms noise for the receiver was 2.2 mV_rms._ The minimum detectable signal (MDS) for the TIA was estimated to be approximately 7.3 mV_rms_ when SNR was 3.3. Because the TIA gain was approximately 76 dBΩ, measured using an electrical pulse response test, the minimum detectable current of TIA was 340 nA. Considering the measured TIA bandwidth of approximately 530 MHz, the input referred noise current could be calculated as 4.48 pA/√Hz.

**Figure 13:**
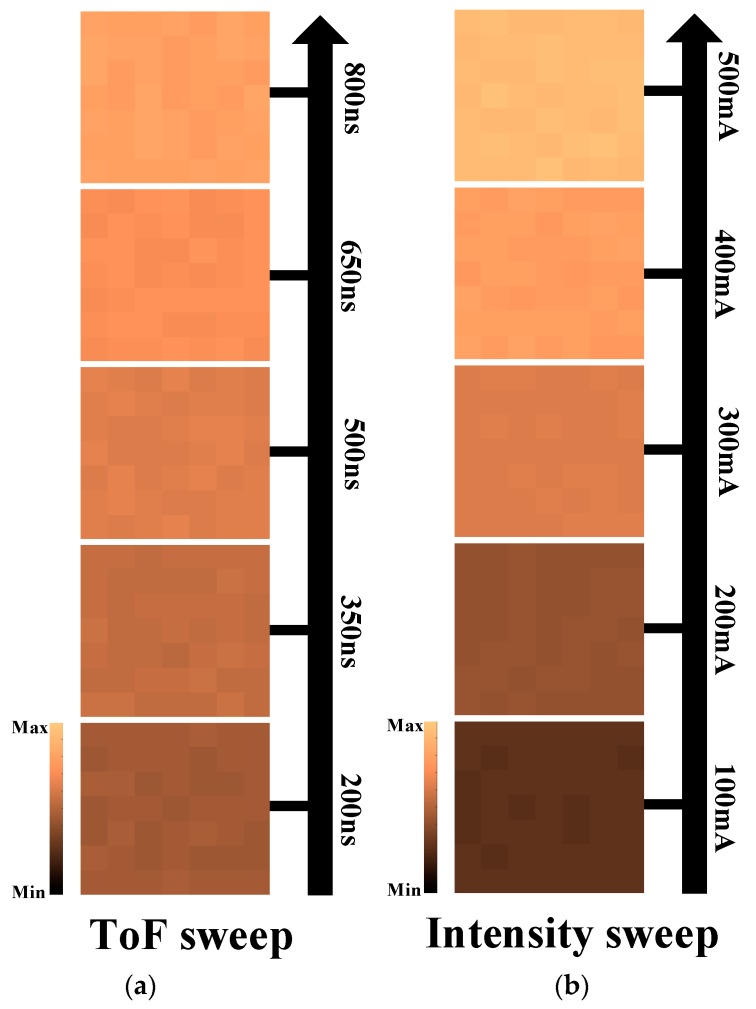
Captured sample images taken from the prototype chip: (**a**) range images with ToF sweep and (**b**) intensity images with laser power sweep.

Figure 13 shows the range and intensity images for the optical pulse response (single-shot measurement) taken from the prototype chip. In order to verify the optical linearity of the prototype chip, the measurement was carried out for both cases: the ToF sweep at a fixed laser pulse amplitude (Figure 13a) and the laser pulse amplitude sweep at a fixed ToF (Figure 13b). The laser power was swept linearly using the bias current control of the laser generator from 100 mA to 500 mA with a minimum step of 100 mA at a ToF of 300 ns. The time interval of the ToF was swept from 300 ns to 700 ns, with a minimum step of 100 ns at the laser bias current of 300mA. From those results, the measured optical non-linearity for the ToF and the intensity was found to be 0.03% and 0.05 % of the full scale, respectively, which is negligible to clarify the echo information.

**Figure 14:**
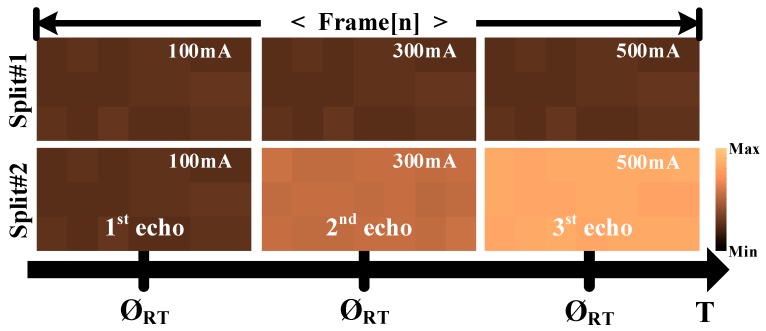
The multiple echo detection capability of the Split#1 and Split#2.

To verify the MTR of the prototype FPA configuration applying the proposed readout scheme, it was assumed that a short laser pulse was emitted directly into the prototype chip by repeated single-shot measurements at specific timing at the Ø_DR_, Ø_SF1_, and Ø_SF2_, with different amplitudes during the Ø_FR_ of 1.25 ms. The captured images of Split#2 were then compared with that of Split#1. As shown in Figure 14, Split#2 collected three intensity images corresponding to the first echo_[n]_ for the Ø_DR_ of 0.625 μS, the second echo_[n−1]_ for the Ø_SF1_ of 0.312 μS, and the third echo_[n−1]_ for the Ø_SF2_ of 0.312 μS, while Split#1 collected only the first echo_[n]_, for the full period of Ø_FR_. Note that Split#1 can only collect the result of the single echo detection. This implies that the MTR of the proposed readout scheme is approximately three times larger than the conventional approach for an FPA-based LADAR structure. Given that the laser pulse generator has a pulse repetition rate of over 100 ns, to further verify the MTR of the prototype receiver, the electrical pulse response measurement was executed and the minimum response interval for the successive incoming echoes was measured as approximately 20 ns.

**Figure 15:**
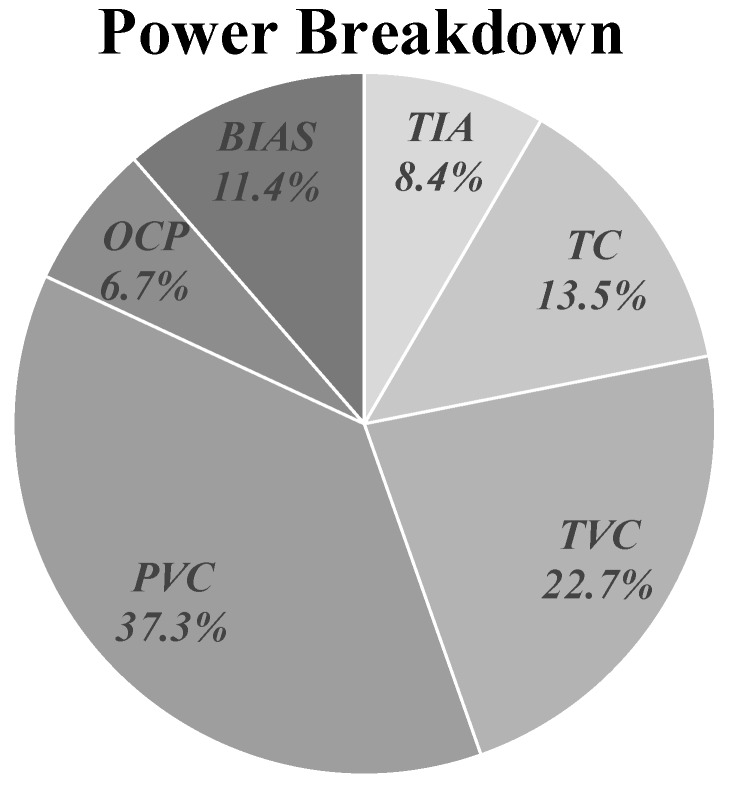
Power breakdown of the prototype LADAR receiver.

Figure 15 shows the simulated power breakdown for the entire prototype FPA-based LADAR receiver. The total averaged power consumption of the proposed multiple echo detection receiver array, in the case of Split#2, was measured as approximately 463 mW, including 54 mW of the output buffer with a supply voltage of 2.8 V. This implied that a single pixel of Split#2, including the APD biasing circuit, dissipated at approximately 7.47 mW. In the case of Split#1 for only the single echo detection structure, a single pixel dissipated at a power of 5.38 mW. When considering the MTR, which was three times larger than that of Split#1, the power consumption of Split#2 can thus be normalized to that of Split#1 as a normalized power consumption of 2.49 mW_N_.

Table 1 shows the performance summary of the prototype LADAR receiver applying the proposed digital-assisted readout scheme in comparison with some recently publish works. The additional echo detection function during the serial data readout period is integrated into the proposed FPA-based LADAR system as a unique function. Assuming that other works applied the FPA configuration, thus forming a basis of comparison, our prototype with the proposed readout scheme showed a three times larger MTR than other works. In addition, the proposed unit LADAR receiver in the FPA-based configuration demonstrated competitive performances compared with recently published papers, even though it consumes less power and is smaller than the others, in spite of the 8 × 8 FPA configuration.

## 5. Conclusions

This work introduces a new FPA-based LADAR receiver architecture designed to utilize the time period of the serial data readout period to enable multiple echo range detection, while also capturing intensity information. The proposed digital-assisted readout scheme allowed the prototype to demonstrate an MTR three times larger than that of a conventional approach, while maintaining a low power consumption and small FPA configuration area. The proposed readout scheme could be a promising solution for high-resolution FPA-based LADAR systems. It can potentially relieve the structural limitations inherent in FPA-based readout architecture.

## Figures and Tables

**Table 1:** Performance comparison.

		This Work	TCAS-I’14 [11] H.-S. Cho	TCAS-I’17 [24] S. Kurtti	Sensors’18 [14] C.-R. Hong	Commercial [25]
**Type**		Integrated	Integrated	Integrated	Integrated	Hybrid
**Technology**		CMOS 0.18 μm	CMOS 0.35 μm	CMOS 0.35 μm	CMOS 0.18 μm	N/A
**PD**	**Type**	InGaAsAPD	InGaAsAPD	N/A	InGaAsPIN-PD	InGaAs APD
	**C_PD_**	1 pF	2.5-5 pF	1.5 pF	0.5 pF	<5pF
	**Wavelength**	1550 nm	1550 nm	905 nm	1550 nm	1550 nm
**Pulse specification**		5 ns	5 ns	3 ns	4 ns	10–28 ns
**Integrated receiver number**		8 × 8	1	1	1 × 12	1
**Intensity capture**		Yes	Yes	Yes	No	No
**Bandwidth**		530 MHz	160 MHz	230 MHz	720 MHz	N/A
**Input referred noise**		4.48 pA/√Hz	1.36 pA/√Hz	6.59 pA/√Hz	6.3 pA/√Hz	N/A
**Power consumption**		Total: 430 mW Unit: 7.47 mW	79 mW	150 mW	Total: 340 mW Unit: 29.8 mW	420 mW
**Current MDS**		340 nA	53 nA	333 nA	1.14 uA	N/A
**Dynamic range**		1:850,	1:12,000	1:100,000	1:11,760	1:100,000
**Walk error**		2.2 n	2.2–2.8 ns	2.5 ns	N/A	N/A
**MTR**	**Single receiver**	20 ns	50 ns	N/A	40 ns	67 ns
	**Applied in FPA**	× 3	× 1	× 1	× 1	× 1
**Chip size**		Total: 2.2 mm^2^	TIA: 1.0 × 1.2 mm^2^	Unit: 2.0 mm^2^	Total: 5.0 × 1.0 mm^2^	N/A
		Unit: 100 μm^2^	Peri.: 1.2 × 1.3 mm^2^			

* Note that the minimal detectable signal (MDS) is normalized with an SNR of 3.3 for comparison.
